# Pediatric Patients Who Underwent Elbow Arthroscopy Had an 86% Return-to-Sport Rate, a 12% Reoperation Rate, and a 3.7% Complication Rate

**DOI:** 10.1016/j.asmr.2024.100952

**Published:** 2024-05-24

**Authors:** Nick F.J. Hilgersom, Myrthe Nagel, Bertram The, Michel P.J. van den Bekerom, Denise Eygendaal

**Affiliations:** a Department of Orthopaedics and Sports Medicine, Erasmus MC, University Medical Center, Rotterdam, The Netherlands; b Department of Orthopaedic Surgery, Amphia Hospital, Breda, The Netherlands; c Department of Orthopaedic Surgery, Onze Lieve Vrouwe Gasthuis, Amsterdam, The Netherlands; d Department of Human Movement Sciences, Faculty of Behavioral and Movement Sciences, Vrije Universiteit, Amsterdam, The Netherlands

## Abstract

**Purpose:**

To assess the applicability and safety of elbow arthroscopy in the pediatric population at our institution by analyzing the indications and complications in a large pediatric patient series.

**Methods:**

We retrospectively identified all patients who underwent elbow arthroscopy at age 18 years or younger from 2006 to 2017 performed by a single fellowship-trained surgeon. The exclusion criteria were follow-up shorter than 8 weeks and open surgical procedures (not fully arthroscopic). Medical records were reviewed for baseline characteristics, indications for elbow arthroscopy, range of motion, complications, and reoperations.

**Results:**

In total, 191 patients (64 boys and 127 girls) were included, with a median age of 15.5 years (interquartile range, 14.0-16.7 years). Indications for arthroscopic surgery were grouped into treatment of osteochondritis dissecans (60%), debridement for bony or soft-tissue pathology (35%), contracture release (3%), and diagnostic arthroscopy (3%). The complication rate was 3.7%, including 4 minor complications (3 superficial wound problems and 1 case of transient ulnar neuropathy) and 3 major complications (1 case of manipulation under anesthesia for stiffness, 1 deep infection, and 1 [unplanned] reoperation for persistent locking within 1 year of the index procedure). Subsequent surgery was required in 23 patients (12%) because of newly developed, persisting or recurring elbow problems. Of the patients, 86% were able to return to sports.

**Conclusions:**

Pediatric elbow arthroscopy performed by an experienced surgeon using a standardized technique for a wide variety of elbow conditions has an acceptable complication rate that is similar to rates in the previously published literature on elbow arthroscopy in the pediatric and adult populations; however, a significant proportion of patients needed subsequent surgery in the following years.

**Level of Evidence:**

Level IV, therapeutic case series.

Classically, elbow arthroscopies in the pediatric population were indicated for the treatment of osteochondritis dissecans (OCD). Both Micheli et al.^1^—who first reported on indications for pediatric elbow arthroscopy—and, more recently, Vavken et al.^2^ showed that 58% of their respective pediatric elbow arthroscopy cases were indicated for the treatment of OCD. However, as elbow arthroscopy is evolving, so are its indications, and a shift toward arthroscopic treatment of acute trauma and arthrofibrosis in the pediatric elbow seems to be occurring.^2^^,^^3^ Andelman et al.^3^ reported on 64 elbow arthroscopies in children, of which 45% were indicated for contracture release, followed by arthroscopically assisted reduction and fixation of elbow fractures (20%) and treatment of OCD (20%). Other reported indications for elbow arthroscopy in the pediatric patient are septic arthritis, debridement of synovitis or posterior impingement, correction of supracondylar fracture malunion, treatment of congenital or perinatally acquired disorders (including cerebral palsy), or solely diagnostic purposes.^1^, ^2^, ^3^, ^4^, ^5^, ^6^, ^7^

The benefits of elbow arthroscopy over open surgery in the pediatric patient are enhanced intra-articular visualization, avoidance of postoperative morbidity associated with large dissections, possibility of planning staged surgical procedures, and faster rehabilitation.^2^^,^^3^^,^^8^ A recent systematic review performed by de Klerk et al.^9^ showed similar complication rates for elbow arthroscopy in the pediatric population (median, 1%; 95% confidence interval, 0%-3.5%) and adult population (median, 0%; 95% confidence interval, 0%-0.4%), despite the narrower anatomic spatial relations in the pediatric elbow. Nonetheless, surgery around the elbow remains prone to serious complications because of the dense and close proximity of nerves to the elbow joint.

Pediatric elbow arthroscopy is incorporating the lessons learned and technical advancements made in the adult population, allowing the procedure to be safe and indications to broaden. Despite these advancements, the applicability of elbow arthroscopy in the pediatric population has remained largely underexposed and partly unexplored. The purpose of this study was to assess the applicability and safety of elbow arthroscopy in the pediatric population at our institution by analyzing the indications and complications in a large pediatric patient series. We hypothesized that pediatric elbow arthroscopy at our institution would be used to treat a variety of elbow conditions and would have a similar safety profile to the currently published literature.

## Methods

Our institutional review board approved this retrospective study of a consecutive series of elbow arthroscopies (No. N2018-0132). The surgical database of a single surgeon (D.E.) with fellowship training in elbow surgery at a tertiary referral center was searched by procedural code for all patients who underwent elbow arthroscopy at age 18 years or younger from 2006 to 2017. The exclusion criteria were follow-up shorter than 8 weeks and open surgical procedures (not fully arthroscopic). Demographic characteristics, laterality, indications for elbow arthroscopy, complications, reoperations, range of motion, and return to sports were collected from medical records.

For analysis of indications, 4 groups were defined: (1) treatment of OCD (OCD group), (2) debridement for bony or soft-tissue pathology (debridement group), (3) contracture release (contracture group), and (4) diagnostic arthroscopy (diagnostic group). Complications were categorized as minor, defined as those that resolved with conservative treatment (e.g., transient nerve injury and superficial wound problems), or major, defined as those that required further surgical intervention within 1 year of the index procedure or led to prolonged disability (e.g., permanent nerve injury or deep infection) attributable to the arthroscopic procedure.^10^ Range of motion was routinely assessed by the senior author (D.E.) at 8 weeks postoperatively, which was also the minimum clinical follow-up period in this series. Medical records were reviewed until the latest date of entry. At the start of the investigated period, the senior author (D.E.) had 6 years of experience in elbow arthroscopy and had treated approximately an additional 30 adult patients per year using elbow arthroscopy.

### Surgical Technique

**Fig 1:**
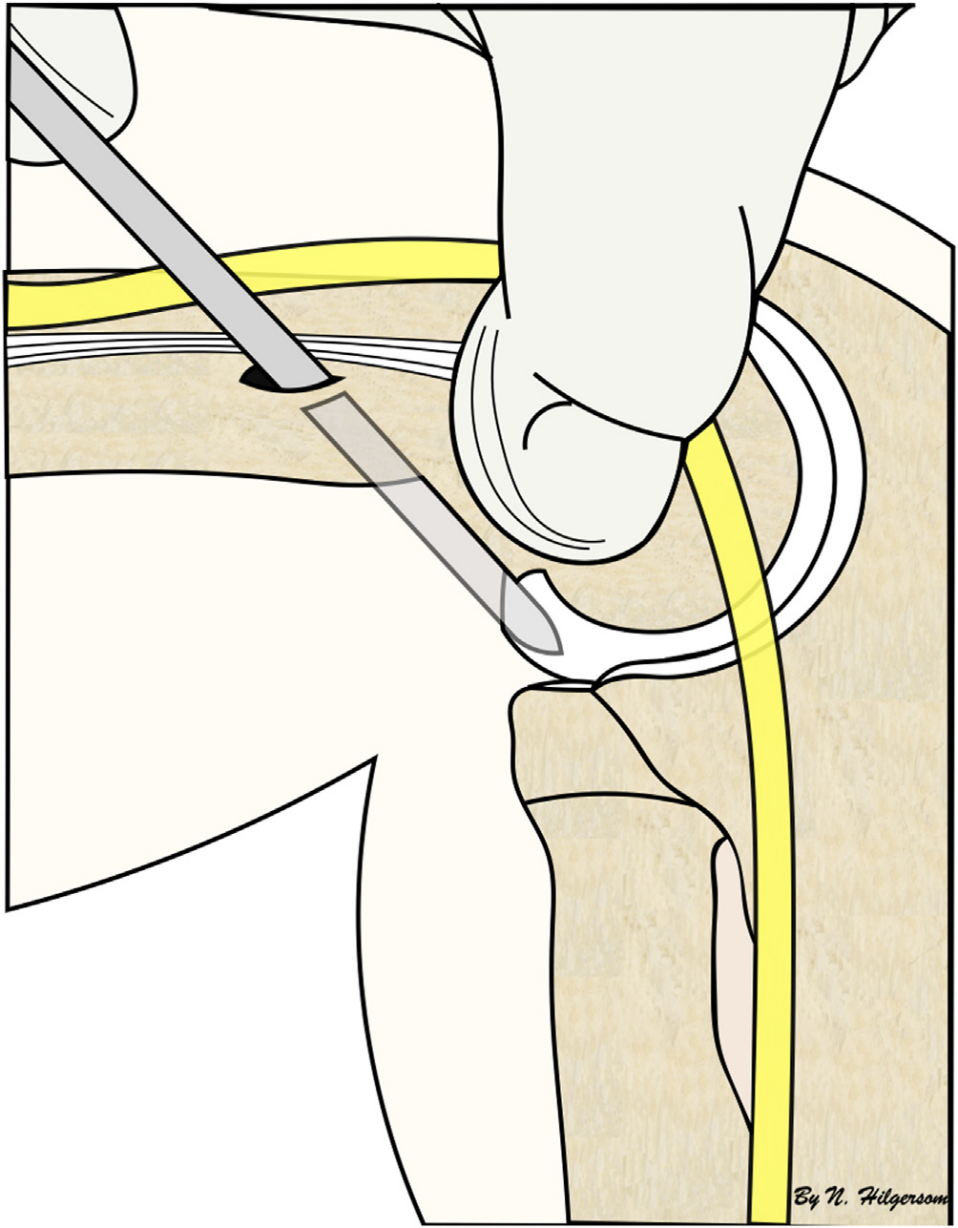
The surgeon determines the location of the ulnar nerve prior to the creation of the proximal anteromedial portal and uses his or her thumb to prevent the nerve from migrating anteriorly while introducing a trocar into the anterior compartment. A right elbow is shown with the patient in the lateral decubitus position.

All procedures were performed in standardized fashion with patients under general anesthesia. Antibiotic prophylaxis (cefazolin) was routinely administered in all procedures. Patients were positioned in the lateral decubitus position on a bean bag with a tourniquet around the upper arm. The upper arm was placed in a padded support allowing full range of motion and adequate space for anterior-compartment visualization. Prior to surgery, the course of the ulnar nerve and relevant (bony) landmarks and portals were palpated and marked (Fig 1), and the elbow was tested for passive range of motion and stability.

When the surgical procedure began, the arm was wrapped using a non-elastic bandage and the tourniquet was insufflated to 200 mm Hg. Next, joint insufflation was performed via a direct posterior injection of saline solution. For visualization of the elbow joint, a standard 30° wide-angle scope (5 mm in diameter) was used. Debridement of bony and soft-tissue pathology was performed using a 4.5- or 2.5-mm shaver. A pump with flow and pressure controls was used for joint irrigation, with a set maximum pressure of 35 mm Hg and low or mid flow.

All arthroscopic procedures started in the posterior compartment. The posterior compartment was approached using a direct posterior viewing portal and a posterolateral working portal. The posterolateral compartment was approached through the aforementioned direct posterior viewing portal and an additional soft-spot working portal. When indicated, a second soft-spot portal was made as a viewing portal. The anterior compartment was approached using a proximal anteromedial viewing portal created with sharp incision of the skin only and an anterolateral working portal created with an outside-in needle technique under direct visualization.^11^ Accessory portals were created when deemed necessary. The proximal anteromedial portal is the preferred starting portal for anterior-compartment visualization to reduce the risk of nerve injury based on studies by Verhaar et al.^12^ and Cushing et al.^13^ Standard portals used during arthroscopy included proximal anteromedial, anterolateral, midlateral or soft-spot, direct posterior, and posterolateral portals. The total number of portals (minimum, 2; maximum, 6) used during arthroscopy varied according to the indication for arthroscopy. Portal sites were closed using nonresorbable transcutaneous suture, and a compressive dressing was applied before tourniquet deflation.

### Statistical Analysis

Baseline characteristics were summarized as absolute numbers with frequencies for categorical variables and as medians with interquartile ranges (IQRs) for continuous variables because data were nonparametrically distributed. Baseline characteristics (age, male sex, affected elbow, and involvement of dominant elbow) were compared between indication groups using the Kruskal-Wallis test. The Wilcoxon signed rank test was performed to compare preoperative and postoperative range of motion. *P* < .05 was considered statistically significant. Statistical analysis was performed STATA software (version 14; StataCorp, College Station, TX).

## Results

### Demographic Characteristics

Medical records were collected from 206 pediatric patients who underwent elbow arthroscopy during the investigated period. Of these patients, 15 (7.3%) were excluded: 7 underwent follow-up at a different hospital, closer to home, because of travel distance, and 8 had insufficient follow-up or were lost to follow-up. Hence, 191 pediatric patients were included. There were 64 boys (34%) and 127 girls (66%), with a median age of 15.5 years (range, 10.0-18.0 years) at the time of surgery. A summary of patient characteristics is shown in Table 1. In total, 181 patients (95%) participated in sports, most of whom participated in overhead sports (71%). The most practiced sport was gymnastics (26%). There were 46 patients (24%) who had sustained a previous fracture of the affected elbow. The most commonly sustained fractures were supracondylar fractures (22%) and radial head fractures (22%). In 23 patients (12%), previous surgery had been performed on the affected elbow (Table 2).

**Table 1:** Demographic Characteristics

Characteristic	Data (N = 191)
Age, median (IQR), yr	15.5 (14.0-16.7)
Male sex	64 (34)
Involved side	
Right	124 (65)
Left	67 (35)
Dominant arm affected	122 (64)
Sport participation	
Gymnastics	50 (26)
Tennis	18 (9)
Field hockey	16 (8)
Soccer	13 (7)
Judo	13 (7)
Horse riding	10 (5)
Biking (cycling, mountain biking)	8 (4)
Fighting sports	8 (4)
Volleyball	8 (4)
Dancing	6 (3)
Athletics	4 (2)
Handball	3 (2)
Baseball	3 (2)
Other sport	21 (11)
No sport	10 (5)
Overhead sports	128 (67)
Sports level (n = 140)	
Recreational	45 (32)
Competitive	67 (48)
Professional	28 (20)

NOTE. Data are presented as number (percentage) unless otherwise indicated.

IQR, interquartile range.

**Table 2:** Previous Surgical Procedures and Fractures

	Data (N = 191)
Previous surgery on involved elbow	23 (12)
Arthroscopic debridement with or without removal of loose bodies	9 of 23 (39)
Arthroscopic debridement and removal of loose bodies plus open OCD fragment fixation	1 of 23 (4)
Open debridement	3 of 23 (13)
Open arthrolysis with or without removal of loose bodies	2 of 23 (9)
ORIF	5 of 23 (22)
ORIF plus arthroscopic arthrolysis	1 of 23 (4)
ORIF plus open debridement	1 of 23 (4)
ORIF plus MCL reconstruction	1 of 23 (4)
	
Previous fracture of involved elbow	46 (24)
Supracondylar fracture	10 of 46 (22)
Intercondylar fracture	4 of 46 (9)
Lateral condyle fracture	2 of 46 (4)
Medial epicondyle fracture (apophysiolysis)	2 of 46 (4)
MCL avulsion	3 of 46 (7)
Capitellar fracture	1 of 46 (2)
Elbow luxation	8 of 46 (17)
Radial head fracture	10 of 46 (22)
Coronoid fracture	1 of 46 (2)
Olecranon fracture	1 of 46 (2)
Antebrachium fracture	1 of 46 (2)
Not specified	3 of 46 (7)

NOTE. Data are presented as number (percentage).

MCL, medial collateral ligament; OCD, osteochondritis dissecans; ORIF, open reduction and internal fixation.

### Indications

The distributions of indications among the indication groups and baseline characteristics per indication group are shown in Tables 3 and 4, respectively. Comparison of baseline characteristics among the groups showed that the debridement group was significantly older than the OCD group, with a median age at the time of surgery of 15.8 years versus 15.1 years (*P* = .002). Furthermore, the dominant elbow was more frequently involved in the OCD group than in the debridement group: 72% versus 51% (*P* = .010).

**Table 3:** Indications

Indication for Surgery	Data (N = 191)
OCD	114 (59.7)
Capitellar	108 (56.5)
Trochlear	4 (2.1)
Radial head	1 (0.5)
Debridement	66 (34.6)
Posterolateral impingement (including synovial fringe and annular hypertrophy)	23 (12.0)
Posterior impingement (including posteromedial impingement and valgus extension overload)	19 (10.0)
Anterior impingement	1 (0.5)
Synovial chondromatosis	1 (0.5)
Trochlear AVN (including fishtail deformity)	13 (6.8)
Post-trauma sequelae (synovitis or loose bodies)	9 (4.7)
Contracture	6 (3.1)
Diagnostic	5 (2.6)
Suspicion of PLRI	1 (0.5)
Suspicion of PRUJ and/or annular ligament instability	1 (0.5)
Locking and catching	1 (0.5)
Pain because of synovitis due to MCL insufficiency	1 (0.5)
Pain because of synovitis	1 (0.5)

NOTE. Data are presented as number (percentage).

AVN, avascular necrosis; MCL, medial collateral ligament; OCD, osteochondritis dissecans; PLRI, posterolateral rotatory instability; PRUJ, proximal radioulnar joint.

**Table 4:** Baseline Characteristics, Complications, and Subsequent Surgical Procedures

	OCD	Debridement	Contracture	Diagnostic
Patients, n	114	66	6	5
Median age (IQR), yr	15.1 (13.4-16.2)∗	15.8 (14.7-17.3)∗	16.5 (14.9-15.9)	15.5 (14.9-15.9)
Male sex	45 (39)	17 (26)	1 (17)	1 (20)
Right elbow	82 (72)	36 (55)	3 (50)	3 (60)
Dominant elbow	82 (72)†	33 (50)†	3 (50)	4 (80)
Previous fracture	8 (7)	31 (47)	6 (100)	1 (20)
Previous surgery	9 (8)	12 (18)	2 (33)	0 (0)
Complications	5 (4): superficial wound problem in 2, deep infection in 1, transient ulnar neurapraxia in 1, and reoperation because of locking in 1	2 (3): superficial wound problem in 1 and MUA because of flexion contracture in 1	0 (0)	0 (0)
Subsequent surgery	11 (10)	10 (15)	0 (0)	1 (20)

NOTE. Data are presented as number (percentage) unless otherwise indicated.

IQR, interquartile range; MUA, manipulation under anesthesia; OCD, osteochondritis dissecans.

∗ Significant difference after pair-wise comparison between OCD and debridement groups: *P* = .002.

† Significant difference after pair-wise comparison between OCD and debridement groups: *P* = .010.

OCD was treated by debridement with the addition of microfracturing in select cases (n = 39) and removal of loose bodies if present. The great majority comprised capitellar OCD (96%); trochlear OCD (4%) and radial head OCD (1%) were also observed. Of 114 patients, 14 (12%) had undergone previous treatment of an OCD lesion of the contralateral elbow. Of these 14 patients, 8 were gymnasts. The remaining 6 patients participated in volleyball (2), judo (2), soccer (1), and scouting (1).

The most common indication in the debridement group was posterolateral impingement (35%), including annular hypertrophy and synovial fringe. The second most common indication was posterior impingement (29%), including posteromedial impingement or valgus extension overload, followed by avascular necrosis (AVN) of the trochlea (20%) and post-traumatic sequelae (14%). A significant number of patients had sustained previous trauma to the operated elbow (47%), most commonly supracondylar or intercondylar fractures (n = 9), followed by radial head fractures (n = 6) and elbow luxation (n = 5). Of 13 patients with trochlear AVN, 10 had a traumatic cause for the development of AVN of the trochlea and presented at a median of 4 years (IQR, 1-7 years) after injury. In 1 case, postseptic arthritis had occurred in infancy. In 2 patients, no clear cause was identified. Previous surgery was performed in 4 of 13 patients: 1 because of a fracture (distal humeral fracture treated with K-wire fixation) and 3 because of trochlear AVN.

The contracture group consisted completely of post-traumatic joint contractures. All patients experienced a previous fracture and/or surgical procedure, consisting of a capitellar fracture for which K-wire fixation was performed (n = 1), a radial head fracture (n = 1), elbow luxation (n = 1), an intercondylar fracture for which open reduction and internal fixation were performed (n = 1), and supracondylar fractures (n = 2). The median preoperative range of motion with flexion-extension in this group was 108° (IQR, 80°-120°); this improved significantly, with a median improvement of 23° (IQR, 15°-30°), resulting in a median postoperative range of motion of 127° (IQR, 120°-140°) (*P* = .028). Diagnostic arthroscopy was used mainly to assess elbow stability (posterolateral rotatory instability, annular ligament instability, and valgus instability) or to treat persistent synovitis despite nonsurgical treatment and the need for arthroscopic synovectomy and synovial biopsy.

### Complications

Among 191 elbow arthroscopy procedures, 7 complications (3.7%) were noted, comprising 4 minor complications (2.1%) and 3 major complications (1.6%) (Table 5). Minor complications included persistent portal site leakage in 2 patients, superficial cellulitis in 1, and transient ulnar neurapraxia in 1. All 3 superficial wound problems recovered completely with conservative treatment. In a 14-year-old gymnast treated for capitellar OCD, transient ulnar neuropathy developed with both sensory symptoms (numb sensation of digits 4 and 5) and motor symptoms (weakness of finger spreaders) that resolved spontaneously within 3 months of surgery. This could most likely be attributed to manipulation or traction of the ulnar nerve with use of the proximal anteromedial portal.

**Table 5:** Complications and Subsequent Surgical Procedures

	Data (N = 191)
Complications	7 (3.7)
Minor	4 (2.1)
Superficial wound problems	3 (1.6)
Transient ulnar neuropathy	1 (0.5)
Major	3 (1.6)
Deep infection	1 (0.5)
Flexion contracture requiring MUA	1 (0.5)
Unplanned reoperation: locking and catching due to loose body	1 (0.5)
Subsequent surgery	23 (12)
OCD: capitellar (persisting symptoms with locking and catching [6] or without locking and catching [6])	12 (52.2)
Debridement	10 (43.4)
Posterolateral impingement (persisting or recurring symptoms)	3 (13.0)
Posterior impingement (recurring symptoms, 1 after new fall)	2 (8.7)
Synovial chondromatosis (recurring symptoms)	1 (4.3)
Trochlear AVN (persisting or recurring symptoms)	3 (13.0)
Post-traumatic (ulnar neuropathy due to luxation of ulnar nerve and snapping triceps)	1 (4.3)
Diagnostic (PRUJ instability for which plication was performed)	1 (4.3)

NOTE. Data are presented as number (percentage).

AVN, avascular necrosis; MUA, manipulation under anesthesia; OCD, osteochondritis dissecans; PRUJ, proximal radioulnar joint.

Major complications include 1 deep infection, 1 case of manipulation under anesthesia for stiffness, and 1 unplanned reoperation. In a 15-year-old male patient who underwent treatment for capitellar OCD, a deep infection developed and was treated by needle aspiration (load reduction) and antibiotics over a course of 6 weeks (flucloxacillin intravenously for 2 weeks, followed by clindamycin orally for 4 weeks). The patient received prophylactic antibiotics at surgery, and no other risk factors could be identified. Intra-articular fluid was aspirated during surgery for microbiological analysis, but unfortunately, the samples were lost due to a logistical error. A 15-year-old girl who was treated for posterior impingement after prior supracondylar fracture by debridement underwent manipulation under anesthesia 2 months postoperatively because of a loss of flexion (range of flexion-extension; 140°-30°-0° preoperatively, 100°-10°-10° postoperatively, and 130°-10°-0° at final follow-up). The probable cause was lack of adherence to the physiotherapy protocol. Pain was well under control after primary surgery. There were no other cases in this series in which impairment of range of motion was an issue, with an overall median postoperative range of motion with flexion-extension of 140° (IQR, 133°-140°). In a 13-year-old girl treated for capitellar OCD, new complaints of locking and catching developed owing to a loose body; she underwent re-arthroscopy 4 months after primary surgery.

There were no cases of vascular injury, permanent nerve injury, deep venous thrombosis, pulmonary embolism, or compartment syndrome. All the minor complications resolved spontaneously, and none of the complications caused persistent disability. Statistical analysis showed no significant difference in the number of complications between the indication groups.

### Subsequent Surgical Procedures

In 23 patients (12.0%), subsequent surgery was required at a median of 2 years (IQR, 1.4-5.3 years) after the index procedure (Table 5). Most of the subsequent surgical procedures were performed because of recurrent or persisting symptoms in patients previously treated for capitellar OCD (n = 12, 52%), followed by posterolateral impingement (n = 3, 13%) and trochlear AVN (n = 3, 13%).

Two patients needed subsequent surgery within 1 year of the index procedure: The first was the aforementioned 13-year-old girl who underwent re-arthroscopy because of a symptomatic loose body after treatment of capitellar OCD, which was considered a major complication. The second was a 15-year-old boy who underwent step-wise treatment of a snapping elbow owing to annular ligament hypertrophy with interposition. At the index procedure, synovectomy and partial shaving of the annular ligament were performed. The stability of the proximal radioulnar joint was tested using a trocar and deemed stable.^14^ Unfortunately, he had persistent complaints of clicking at 4-month follow-up and therefore subsequently underwent annular ligament reconstruction with triceps autograft (fascial sling) because of proximal radioulnar joint instability.

### Return to Sports

Return to sports could be determined in 119 of 181 patients (66%) (Table 6). In total, 102 patients (86%) were able to return to sports. Seventy-one patients (60%) returned to the same sport and level. Twenty-five patients (21%) returned to the same sport at a lower level or changed sports related to the elbow. Thirteen patients (11%) did not return to sports because of the elbow.

**Table 6:** Return to Sports

	Return to Sports (n = 119)
Yes	
Same sport at same level	71 (60)
Same sport at lower level, related to elbow	14 (12)
Different sport, related to elbow	11 (9)
Different sport, not related to elbow	6 (5)
No	
Related to elbow	13 (11)
Not related to elbow	4 (3)

NOTE. Data are presented as number (percentage).

## Discussion

In the pediatric population at our institution, elbow arthroscopy was performed to treat a variety of pediatric elbow pathology, mainly capitellar OCD, with an acceptable complication rate of 3.7% that is comparable to the available literature.^9^ A significant proportion of patients (12%) needed subsequent surgery in the following years.

The main indication for pediatric elbow arthroscopy at our institution was OCD (60%). This is very similar to previously published series on pediatric elbow arthroscopy by Micheli et al.^1^ and Vavken et al.,^2^ with 55% and 58% of their respective populations undergoing arthroscopy for the treatment of OCD. The wide variety of elbow conditions treated in our study reflects the broad applicability of arthroscopy in the treatment of pediatric elbow pathology. These conditions include posterior, posterolateral, and anterior impingement; OCD; post-traumatic sequelae such as synovitis and loose bodies; and contracture release—in addition to rarer pediatric conditions such as synovial chondromatosis, snapping elbow (annular hypertrophy with or without proximal radioulnar joint instability), and trochlear AVN (including fishtail deformity). The patient cohort studied was treated in a hospital highly specialized in treating upper-limb pathology in children, which contributed to the wide variety of elbow pathology that was seen and treated.

The complication rate in this study was 3.7%, comprising a minor complication rate of 2.1% and major complication rate of 1.6%, which fits well within the range of complication rates reported in the available literature on elbow arthroscopy in the pediatric and adult populations.^9^ Previously published studies on elbow arthroscopy in the pediatric population including treatment of various indications are scarce and have reported complication rates ranging from 0% to 17%.^1^, ^2^, ^3^^,^^15^ Vavken et al.^2^ reported on the first 50 pediatric elbow arthroscopies performed by a single surgeon and found an 8% complication rate, involving only minor complications (3 cases of transient neurapraxia and 1 superficial wound infection). Andelman et al.^3^ reported on 64 pediatric elbow arthroscopies, mainly performed for contracture release and arthroscopically assisted fracture reduction and fixation, and showed a complication rate of 17.2%, with a major complication of 6.3% (1 supracondylar humeral stress fracture and 3 flexion contractures) and minor complication rate of 10.9% (2 superficial wound problems, 2 cases of transient neurapraxia, 1 partial physeal bar, and 1 flexion contracture). Their relatively high complication rate was attributed to the large proportion of patients treated for post-traumatic contracture.

The study by Elfeddali et al.^16^ may be best suited for comparison to the adult population because it was performed at the same institution and the procedures were performed by the same surgeon (D.E.). In a series of 200 elbow arthroscopies, an overall complication rate of 7.5% was determined, comprising a major complication rate of 0.5% (1 ulnar nerve injury) and minor complication rate of 7% (3 cases of transient neurapraxia, 4 superficial wound problems, 6 persistent contractures, and 1 mild increase in contracture). The lower complication rate in our study compared with the study of Elfeddali et al. may be best ascribed to variations in the pediatric and adult elbow pathology treated, in particular the proportion of patients treated for joint contracture with arthroscopic arthrolysis (3% vs 38%), accounting for 7 of 14 complications in their study.

The incidence of nerve injury in this series was low, 0.5%, comprising 1 case of transient ulnar neuropathy. Nerve injuries are the most frequently observed complication after elbow arthroscopy in the adult population, with reported incidence rates between 1.3% and 7.5%, and they most commonly involve the ulnar nerve.^9^^,^^17^ Transient nerve injuries have also been described in the scarce literature on elbow arthroscopy in the pediatric population, with 3 ulnar nerve cases, 2 radial nerve cases, and 1 unspecified case, showing incidence rates of 0% to 6%.^1^, ^2^, ^3^^,^^9^ Although spatial relations in the pediatric elbow are smaller, the incidence rates of nerve injury appear similar between the pediatric population and adult population. Fortunately, the vast majority of nerve injuries are transient; however, permanent nerve injuries do occur and have serious debilitating consequences for the patient. To prevent nerve injuries, a standardized approach is paramount, including adhering to basic principles such as marking anatomic landmarks, palpating the course of the ulnar nerve, performing joint distension, and using proximal anteromedial and/or anterolateral portals.^11^ The incidence of ulnar nerve luxation or subluxation in children is higher than that in adults, with reported incidence rates of up to 37%^18^, ^19^, ^20^ versus 21%,^21^^,^^22^ which underlines the importance of determining the location of the ulnar nerve prior to creation of an anteromedial portal by palpation or mini-incision to prevent injury (Fig 1).

The most commonly observed complications in this series were of an infectious nature, with an incidence of 2.1%, comprising 3 minor complications (persistent portal site drainage in 2 cases and superficial cellulitis in 1 case) and 1 major complication (deep infection). Vavken et al.^2^ and Andelman et al.^3^ have reported similar incidence rates in the pediatric population, with 2% and 3.1% rates of superficial wound infection and/or persistent wound drainage, respectively. Intravia et al.^23^ analyzed the complications of 560 elbow arthroscopies performed in a community-based practice by various surgeons. Of 560 procedures, 113 were performed in pediatric patients. Overall, they reported a 2% superficial infection rate and 0.5% deep infection rate. Similar incidence rates of infectious complications have been reported in large series in the adult population, with 2% to 6.9% superficial infection rates and 0% to 2.2% deep infection rates.^10^^,^^16^^,^^24^^,^^25^ To reduce the risk of infection, the patients’ arms were wrapped using a non-elastic bandage during surgery to minimize soft-tissue swelling and the portal sites were closed with nonresorbable sutures in this series. In addition, all patients received prophylactic antibiotics according to surgeon preference. The routine administration of prophylactic antibiotics is debatable regarding its effectiveness in preventing infectious complications during arthroscopy in the pediatric population, its cost-effectiveness, and the prevention of the rise of drug-resistant organisms.^10^^,^^26^^,^^27^ No specific causes of infectious complications could be identified in this series. One could reason that compression of soft tissues at the portal site by the camera or instruments when working at a difficult angle for a prolonged time could lead to wound healing problems.

In total, 23 patients (12%) needed subsequent surgery after the index procedure. Most of these reoperations, 17 of 23 (74%), were performed for chronic pediatric elbow conditions that relatively often require multiple surgical procedures, including synovial chondromatosis, trochlear AVN, and capitellar OCD. Synovial chondromatosis is a rare, benign proliferative condition of the synovial membrane that can be treated by arthroscopic synovectomy and removal of loose bodies with a significant risk of recurrence and reoperation rate of up to 18%.^28^ The pathogenesis of both OCD and trochlear AVN is incompletely understood, but both involve disturbance of the vascular supply to the physis, subchondral bone, and/or cartilage in the skeletally immature patient.^29^^,^^30^ Glotzbecker et al.^31^ reported on 7 patients treated for trochlear AVN with arthroscopic debridement, of whom 4 (57%) needed subsequent surgery. Reoperation rates for capitellar OCD ranging from 2% to 13% after debridement or microfracturing were reported in a systematic review by McLaughlin et al.^32^ More recently, Rothermich et al.^33^ retrospectively analyzed 90 patients treated for capitellar OCD with a minimum follow-up period of 2 years and found a failure rate of 12%. In our study, 12 patients treated for capitellar OCD at the index procedure needed subsequent surgery, indicating an 11% reoperation rate (12 of 108 patients) in the capitellar OCD group.

Return to sports is an important functional outcome for young patients. How exactly to interpret return-to-sport results, however, is quite troublesome. First, returning to sports or returning to a certain level may be interpreted differently per the individual. Second, the outcome has its shortcomings because, for example, it does not include the absence or presence of elbow complaints or how much time it took patients to return to the same level of sport. Finally, a 100% return-to-sport rate is a perfect outcome, but defining what percentage is good, acceptable, or poor is, in part, arbitrary. Current findings show that 86% of patients were able to return to sports and 60% of patients were able to return to the same sport and level. These findings correspond with the overall reported return-to-sport rates of 74% to 100% and reported rates of return to the same sport and level of 40% to 100% in a systematic review on elbow arthroscopy in the pediatric population by Gouveia at al.^34^ Nonetheless, it is important to point out that 11% of patients quit sports because of the elbow. This finding and the possible need for future surgery (12%) should be discussed during counseling of young patients and their caretakers. The primary strengths of this study are the large number of patients included and the variety of pediatric elbow conditions treated by arthroscopy using standard “adult-sized” arthroscopic instruments.

### Limitations

An important limitation of this study that needs to be discussed is its external validity. First, all procedures in this study were performed by a single experienced surgeon. Elbow arthroscopy is a technically challenging surgical technique with a learning curve, possibly even more so in the pediatric patient with smaller anatomic dimensions. Second, few procedures were performed for some of the conditions, partly inherent to the rarity of some pediatric elbow conditions. An elbow surgeon treating young patients needs to have thorough and specific knowledge of the pathophysiology of and treatment options for pediatric elbow conditions, including common conditions such as OCD and rarer conditions such as snapping elbow and trochlear AVN. Therefore, the current findings show the possible wide applicability of elbow arthroscopy in expert hands but do not necessarily extend to the occasional elbow arthroscopist.

## Conclusions

Pediatric elbow arthroscopy performed by an experienced surgeon using a standardized technique for a wide variety of elbow conditions has an acceptable complication rate that is similar to rates in the previously published literature on elbow arthroscopy in the pediatric and adult populations; however, a significant proportion of patients needed subsequent surgery in the following years.

## Disclosures

The authors report the following potential conflicts of interest or sources of funding: D.E. receives personal fees from AO International as course director and International Bone Research Association (10.13039/100009565IBRA) as course director and speaker, outside the submitted work. All other authors (N.F.J.H., M.N., B.T., M.P.J.V.) declare they have no known competing financial interests or personal relationships that could have appeared to influence the work reported in this paper. Full ICMJE author disclosure forms are available for this article online, as supplementary material.

## Supplementary Data

ICMJE author disclosure forms
